# Cooperation Prevails When Individuals Adjust Their Social Ties

**DOI:** 10.1371/journal.pcbi.0020140

**Published:** 2006-10-20

**Authors:** Francisco C Santos, Jorge M Pacheco, Tom Lenaerts

**Affiliations:** 1 Computer and Decision Engineering Department, Institut de Recherches Interdisciplinaires et de Développements en Intelligence Artificielle, Université Libre de Bruxelles, Brussels, Belgium; 2 Program for Evolutionary Dynamics, Harvard University, Cambridge, Massachusetts, United States of America; 3 Department of Physics of the Faculty of Science, Center for Theoretical and Computational Physics, University of Lisbon, Lisbon, Portugal; 4 SWITCH Laboratory, Flanders Interuniversity Institute for Biotechnology, Vrije Universiteit Brussel, Brussels, Belgium; 5 Department of Computer Science, Vrije Universiteit Brussel, Brussels, Belgium; Northwestern University, United States of America

## Abstract

Conventional evolutionary game theory predicts that natural selection favours the selfish and strong even though cooperative interactions thrive at all levels of organization in living systems. Recent investigations demonstrated that a limiting factor for the evolution of cooperative interactions is the way in which they are organized, cooperators becoming evolutionarily competitive whenever individuals are constrained to interact with few others along the edges of networks with low average connectivity. Despite this insight, the conundrum of cooperation remains since recent empirical data shows that real networks exhibit typically high average connectivity and associated single-to-broad–scale heterogeneity. Here, a computational model is constructed in which individuals are able to self-organize both their strategy and their social ties throughout evolution, based exclusively on their self-interest. We show that the entangled evolution of individual strategy and network structure constitutes a key mechanism for the sustainability of cooperation in social networks. For a given average connectivity of the population, there is a critical value for the ratio *W* between the time scales associated with the evolution of strategy and of structure above which cooperators wipe out defectors. Moreover, the emerging social networks exhibit an overall heterogeneity that accounts very well for the diversity of patterns recently found in acquired data on social networks. Finally, heterogeneity is found to become maximal when *W* reaches its critical value. These results show that simple topological dynamics reflecting the individual capacity for self-organization of social ties can produce realistic networks of high average connectivity with associated single-to-broad–scale heterogeneity. On the other hand, they show that cooperation cannot evolve as a result of “social viscosity” alone in heterogeneous networks with high average connectivity, requiring the additional mechanism of topological co-evolution to ensure the survival of cooperative behaviour.

## Synopsis

In social networks, some individuals interact with more people and more often than others. In this context, one may wonder: under which conditions are social beings willing to be cooperative? Current models proposed in the context of evolutionary game theory cannot explain cooperation in communities with a high average number of social ties. Santos, Pacheco, and Lenaerts show that when individuals are able to simultaneously alter their behaviour and their social ties, cooperation may prevail. Moreover, the structure of the final networks corresponds to those found in empirical data. Their article concludes that the more individuals interact, the more they must be able to promptly adjust their partnerships for cooperation to thrive. Consequently, to understand the occurrence of cooperative behaviour in realistic settings, both the evolution of the complex network of interactions and the evolution of strategies should be taken into account simultaneously.

## Introduction

Conventional evolutionary game theory predicts that natural selection favours the selfish and strong [1], in spite of existing evidence showing that cooperation is more widespread than theory predicts [2]. When cooperation is modelled in terms of the prisoner's dilemma [3] (PD), the solution of the replicator dynamics equation in infinite, well-mixed populations [4–6] dictates the extinction of cooperators by defectors. Cooperators become evolutionarily competitive, however, whenever individuals are constrained to interact with few others along the edges of sparse graphs as recently concluded in two independent studies [7,8]. Both studies place individuals on the nodes of a static graph, and associate their social ties with the vertices linking the nodes such that, throughout evolution, every individual has the possibility of changing her strategy, but not her social ties. In [7] it has been shown that, under strong selection (fitness is determined by the game payoff), heterogeneous graphs lead to a significant increase in the overall survivability of cooperation, modelled in terms of the most popular social dilemmas, played on networks of different degrees of heterogeneity [9]. For the classical PD in which the act of cooperation involves a cost *c* to the provider, resulting in a benefit *b* (*b > c*) for the recipient, a simple relation has been obtained in [8] for a single cooperator to have a chance to survive in a population of defectors, whenever selection is weak (game payoff introduces a small perturbation onto fitness): *b/c > z,* where *z* stands for the average number of ties each individual has (*z* is the average degree of the graph). Both studies show that *games on graphs* open a window for the emergence of cooperation, showing how “social viscosity” alone [8] can contribute to the emergence of cooperation.

However, recent data shows that realistic networks [10–16] exhibit average connectivity values ranging from 2 to 170, with an associated heterogeneity intermediate between single-scale and broad-scale [11], which differs from the connectivity values typically used in previous studies [7,8]. For instance, the network of movie actors exhibits an average connectivity of 30 [17], whereas collaboration networks based on co-authorship of published papers vary from average values of 4 (mathematics), to 9 (physics) up to 15 (biology) [13]. In terms of the simple rule for the evolution of cooperation for graphs, the reported values of *z* require benefits to often exceed costs by more than one order of magnitude for a single cooperator to survive [8]. None of the previous results on strong [7] and weak [8] selection on graphs is capable of explaining how cooperation thrives on such social networks. Other mechanisms have to be at work here that allow for the survival of cooperation.

In most evolutionary models developed so far, social interactions are fixed from the outset. Such immutable social ties, associated naturally with static graphs, imply that individuals have no control over the number, frequency, or duration of their ties; they can only evolve their behavioural strategy. A similar observation can be made on studies related to the physical properties of complex networks [10–16]. The analyzed networks constitute but one static snapshot of networks that have been typically produced by some growth process. Yet, networks have naturally evolved before and will continue to evolve after the snapshot has been taken. Indeed, recent longitudinal studies of evolving social networks [18] indicate that global properties seem to remain rather stable, whereas individual patterns of social ties remain evolving in time. Hence, assuming a fixed population size and global average connectivity, one may ask: *What role do changes in the interaction framework play in the evolution of cooperation* and *to which extent will the social dilemmas influence the topology and heterogeneity of the evolving network*?

Using a minimal model that combines strategy evolution with topological evolution, and in which the requirements of individual cognitive capacities are very small, we investigate under which conditions cooperation may thrive. Network heterogeneity, which now emerges as a result of an entangled co-evolutionary dynamics, will be shown to play a crucial role in facilitating cooperative behaviour.

### A Minimal Co-Evolutionary Model

Let us consider two types of individuals—cooperators and defectors—who engage in several of the most popular social dilemmas of cooperation (see below). They *are not required to accumulate information on all other players,* only those they are immediately connected with. Moreover, they are able to decide, on an equal footing with all players, those ties that they want to maintain and those they want to change. Given an edge with individuals *A* and *B* at the extremes, we say that *A* (*B*) is satisfied with the edge if the strategy of *B* (*A*) is a cooperator, being dissatisfied otherwise. If *A* is satisfied, she will decide to maintain the link. If dissatisfied, then she may compete with *B* to *rewire* the link (as illustrated and described in Figure 1), rewiring being attempted *to a random neighbour of B*. The intuition behind this reasoning is the following: simple agents, being rational individuals with limited information, tend to interact with other agents that are close by in a social manner [18]. In this sense, agent *A* is more likely to encounter one of the friends of *B* and become a friend with *B*'s neighbour. Moreover, selecting a neighbour of an inconvenient partner may turn out to be a good choice, since this partner also tries to establish links with cooperators, making it more likely that the rewiring results in a tie to a cooperator. Indeed, for all social dilemmas described below, a link with a cooperator maximizes the fitness of any individual, irrespective of its (fixed) strategy. Consequently, all individuals naturally seek to establish links with cooperators. Hence, rewiring to a neighbour of a defector is certainly a good choice for individuals with local information only.

**Figure 1:**
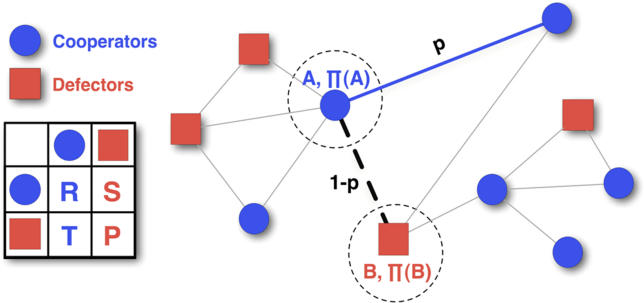
Evolving the Neighbourhood Cooperators and defectors interact via the edges of a graph. B (A) is satisfied (dissatisfied), since A (B) is a cooperator (defector). Therefore, A wants to change the link whereas B doesn't. The action taken is contingent on the fitness Π(A) and Π(B) of A and B, respectively. With probability *p* = [1 + *e* ^−*β*[Π(*A*) − Π(*B*)]^]^−1^ (where *β* = 0.005, (see Materials and Methods)), A redirects the link to a random neighbor of B. With probability 1 − *p,* A stays linked to B. Other possibilities occur: whenever both A and B are satisfied, nothing happens. When both A and B are dissatisfied, rewiring takes place such that the new link keeps attached to A with probability *p* and attached to B with probability 1 − *p*.

The social dilemmas of cooperation examined in this work are modelled in terms of symmetric two-player games, where the players can either cooperate or defect upon interaction. When both cooperate, they receive the payoff *R* (the reward for mutual cooperation). On the other hand, when both defect, they both obtain the payoff *P* (the punishment for mutual defection). The two remaining possibilities occur when one defects and the other cooperates, resulting in the payoff *T* (the temptation to cheat) for the defector and *S* (the disadvantage of being cheated) for the cooperator. Depending on the relative ordering of these four payoff values and assuming that mutual cooperation is preferred over mutual defection, three well-known social dilemmas emerge [9]—the *snowdrift* game (SG) (*T > R > S > P*), the *stag-hunt* game (SH) (*R > T > P > S*) and the *prisoner's dilemma* (PD) (*T > R > P > S*). The dilemma follows from the players' payoff preferences. In the SG game, the players are referred to as greedy since they prefer unilateral defection to mutual cooperation (*T > R*). In the SH game, mutual defection is preferred to unilateral cooperation (*S < P*), resulting in an intrinsic fear for the players to cooperate. Finally, both dilemmas are combined in the PD game, making it the most difficult situation for cooperation to arise. We adopt the convention of [7] and normalize the difference between mutual cooperation (*R*) and mutual defection (*P*) to 1, making *R =* 1 and *P =* 0, respectively. As a consequence, we investigate all dilemmas, summarized in Table 1, in a 2-D parameter space, depicted in Figure 2, where the payoff *T* (temptation to cheat) satisfies 0 *≤ T ≤* 2 and the payoff *S* (disadvantage of being cheated) satisfies *−*1 *≤ S ≤* 1. The *fitness* of each individual corresponds to the total accumulated payoff resulting from pairwise interactions (see Materials and Methods) with all her neighbours.

**Table 1:**
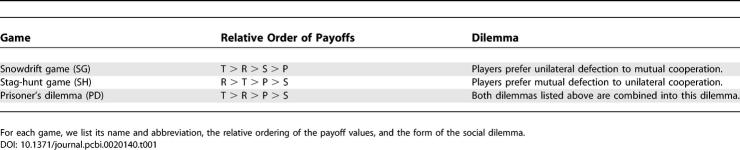
Games Studied and Their Parameter Range

**Figure 2:**
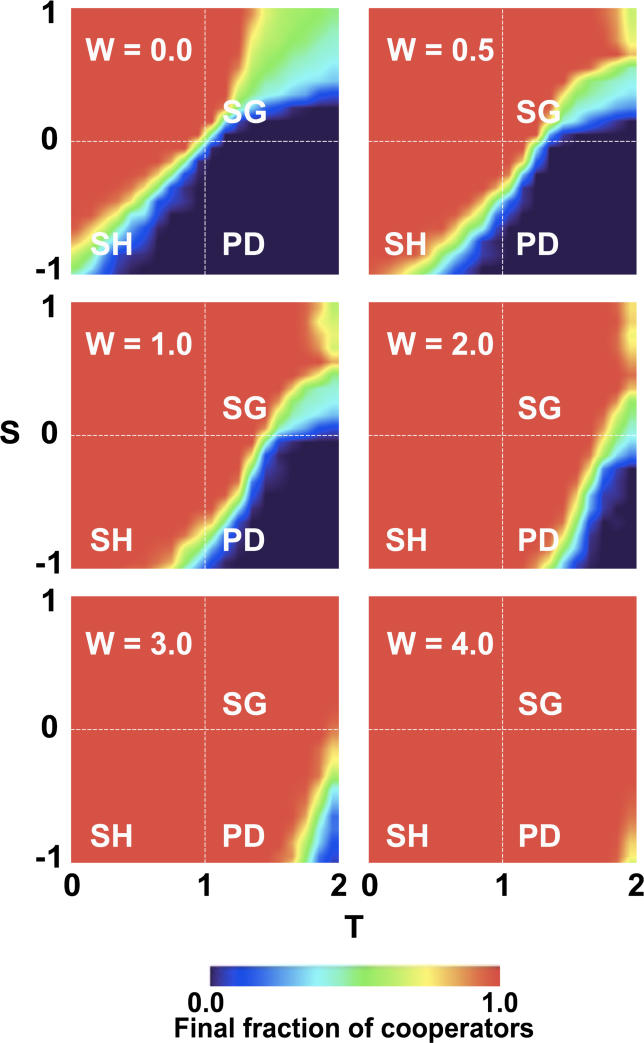
Co-Evolution for Different Dilemmas and Time Scales Results for the fraction of successful evolutionary runs ending in 100% cooperation for different values of the time-scale ratio *W*. We study the most popular social dilemmas of cooperation: the prisoner's dilemma (PD) (*T >* 1 *>* 0 *> S*), the snowdrift game (SG) (*T >* 1*> S >* 0), and the stag-hunt game (SH) (1 *> T >* 0 *> S*). For *W =* 0 (*N =* 10^3^, *z =* 30 and *β =* 0.005), the results fit the predictions from well-mixed populations. With increasing *W* the rate at which individuals readjust their ties increases, and so does the viability of cooperation. Above a critical value (*W_critical_* ≈ 4.0, detailed in Figure 3), cooperators efficiently wipe out defectors. According to [8], only when *b/c >* 30 would cooperation be favored. Co-evolution leads to full cooperation even when *b/c =* 2 (*T =* 2, *S =* −1).

**Figure 3:**
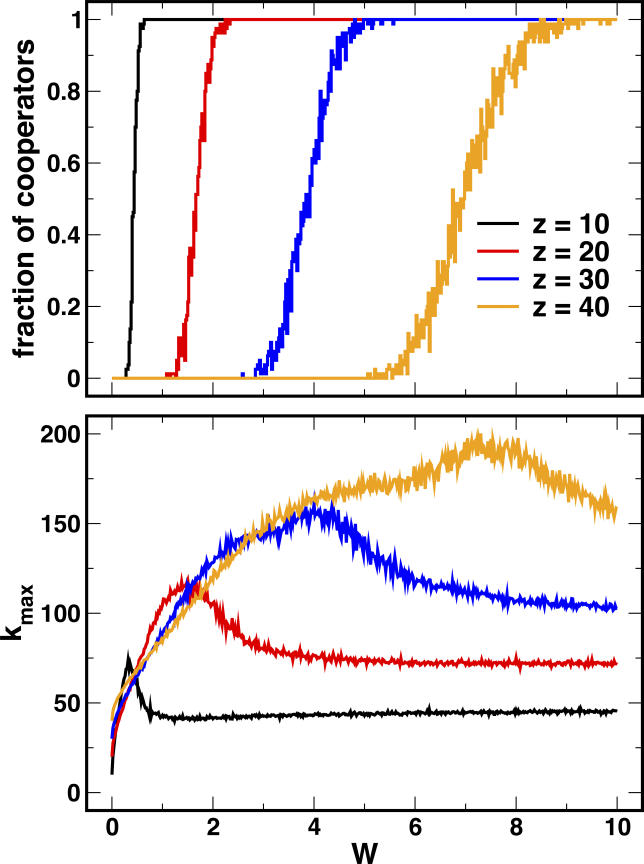
Co-Evolution in the PD for Different Time Scales PD with *b/c =* 2 (*T =* 2,*S = −*1 and *β =* 0.005). (Upper panel) Fraction of cooperators at end as a function of *W* for different values of *z,* each drawn with a different color. For each value of *z,* there is a critical value of *W* − *W_critical _*— above which cooperators wipe out defectors. (Lower panel) Maximum value of the connectivity in population as a function of *W.* With increasing *z, W_critical_* increases. In all cases, the heterogeneity of the associated network becomes maximal at *W_critical_,* stagnating for higher values.

The fact that in our model cooperators and defectors interact via social ties they both decide upon establishes a coupling between individual *strategy* and population *structure*: the game payoff induces now an entangled co-evolution of strategy and structure (for different approaches specific to the PD see [19–22], and to the SH see [23]). Such an *adaptive* individual behaviour introduces a new time scale (*τ_a_*), not necessarily equal to the time scale associated with strategy *evolution* (*τ_e_*). Depending on the ratio *W* = *τ_e_ / τ_a_ ,* different fates may occur for cooperation. Indeed, whenever *W* → 0, we recover the results of [7,8]. On the other hand, with increasing *W,* individuals become apt to adapt their ties with increasing efficiency. In general, however, one expects the two time scales to be of comparable magnitude in realistic situations (see Figures 2 and 3). *W* provides a measure of individuals' inertia to react to their rational choices both at strategy and topological levels: large values of *W* reflect populations in which individuals react promptly to adverse ties, whereas smaller values reflect some overall inertia for topological change (compared with strategy change). In general, the type of response will change from individual to individual. Hence, *W* reflects here an average characteristic of the population.

## Results/Discussion

### Co-Evolution of Strategy and Structure

The contour plots in Figure 2 show how cooperators survive for different values of the ratio *W* in networks with high connectivity. For the most popular social dilemmas of cooperation, we plot the fraction of cooperators who survive evolution, and averaged over 100 independent realizations for the same values of the temptation to cheat *T,* the disadvantage of being cheated *S,* and *W*. For *W* = 0 and moderate selection intensity, tuneable by the *inverse temperature of selection β* (see Materials and Methods), the results reproduce, as expected [7], the predictions for finite, well-mixed populations [7,24] (whereas here *N* = 10^3^ and *z =* 30). Yet, with increasing *W* we observe a “*wave of cooperation*” moving “southeast” towards the lower right corner of each contour plot. Hence, the PD for *T* = 2 and *S* = −1 constitutes the hardest challenge for cooperation within the parameter space studied. Nonetheless, for sufficiently large values of *W,* one obtains as a result of the entangled co-evolution of strategy and structure a *full cooperative scenario*. The swifter the response of individuals to the nature of their ties, the easier it gets for cooperators to wipe out defectors. Similar conclusions have been obtained with different co-evolutionary models, both in the context of the SH game [23], and in the context of the PD game [18,25], supporting the robustness of the current results. Indeed, both models use different rules for structural updating as well as for strategy updating, leading to results in accord with the general findings reported here. Additional insight is provided in Figure 3 (upper panel), where we investigate how cooperation wins over defection as a function of *W* for the PD when *T* = 2 and *S* = −1 (corresponding to *b/c* = 2 in the classical PD) and different values of *z* ∈ [10,40]. For small *W,* cooperators have no chance. Their fate changes as *W* approaches a critical value *W_critical_*—which increases monotonically with *z*—cooperators wiping out defectors above *W_critical_* (the increase of *W_critical_* with *z* is expected, since there are more links to be rewired). Thus, the survival of cooperation relies on the capacity of individuals to adjust to adverse ties, even when the average connectivity is high.

Figure 3 also provides evidence of the detailed interplay between strategy and structure. On the one hand, strategy updating promotes a local assortment of strategies, since cooperators “breed” cooperators and defectors “breed” defectors. On (static) heterogeneous graphs, and for specific values of *T* and *S,* the population will evolve into a cooperative state [7], and the transient dynamics associated with the approach to such a cooperative state, starting from an equal fraction of cooperators and defectors, has been examined in detail in [26]. On the other hand, under structural updating, however, one is promoting local assortative interactions between cooperators and disassortative interactions between defectors and cooperators, which constitute “favourable steps” from an individual point of view. Clearly, when simultaneously active, strategy update will reinforce assortativity among cooperators, but will inhibit disassortativity between defectors and cooperators, which overall will benefit the emergence of cooperation. Furthermore, since for any finite *W* graph heterogeneity will develop as a result of structural update (we are starting from homogenous graphs), it will become easier for strategy update to promote cooperation. Nonetheless, Figure 3 shows that, for small *W,* cooperators become extinct. As *W* approaches *W_critical_ ,* there will be, overall, only a small fraction of surviving cooperators. Under structural update alone, a small number of cooperators will lead to the emergence of strongly heterogeneous networks of ties. This is shown in Figures 3 (lower panel) and 4, where we investigate how the evolved network topology changes with *W,* by plotting the overall network heterogeneity. In the lower panel of Figure 3 we plot the maximum value of the connectivity (*k_max_*) as a function of *W* for *T =* 2 and *S =* −1. For small *W,* heterogeneity remains low since cooperators react slowly to adverse ties, being wiped out. As *W* approaches *W_critical_,* heterogeneity develops, exhibiting a peak at ≈*W_critical_,* where the interplay between strategy and structure is maximal. Finally, above *W_critical_,* heterogeneity decreases, since individual rewiring is fast enough to wipe out defectors. Indeed, when *W* ~ *W_critical_ ,* the small fraction of surviving cooperators promotes them into natural hubs, since they attract links from a neighbourhood whose structure is always evolving due to the sizeable role played by strategy dynamics. Such an entangled co-evolution results in highly heterogeneous graphs. In other words, for *W* ~ *W_critical_,* structural dynamics prevents cooperators from becoming extinct, promoting an underlying heterogeneous network of contacts which provides a favourable topology for cooperation to thrive under strategy dynamics. With increasing *W,* the structural dynamics is fast enough to prevent the fraction of cooperators to be reduced to residual values. More cooperators, in turn, means more competition at sites for rewiring, which leads to a reduction of the overall network heterogeneity as compared with that obtained for *W_critical_*.

For any *W >* 0, individual choices lead to heterogeneous graphs in which some individuals interact more, and more often than, others. In view of the results shown in Figure 2, we show in the top-left panel of Figure 4 the cumulative degree distributions (see Materials and Methods) for the most difficult challenge to cooperation in our parameter space: the PD with (*T =* 2,*S =* −1), corresponding to *b/c =* 2. Starting from a distribution exhibiting a sharp cutoff at *k_max_ = z,* as soon as *W >* 0, the distribution widens, reflecting the increase in heterogeneity of the pattern of ties in the population, where one observes both single-scale and broad-scale heterogeneities in accord with empirical evidence [11]. As shown explicitly in the contours of Figure 4, the amount of heterogeneity depends on the underlying social dilemma. In other words, different challenges to cooperation lead to the evolution of different societal organization, in which simple-to-broad–scale heterogeneity is ubiquitous [11].

**Figure 4:**
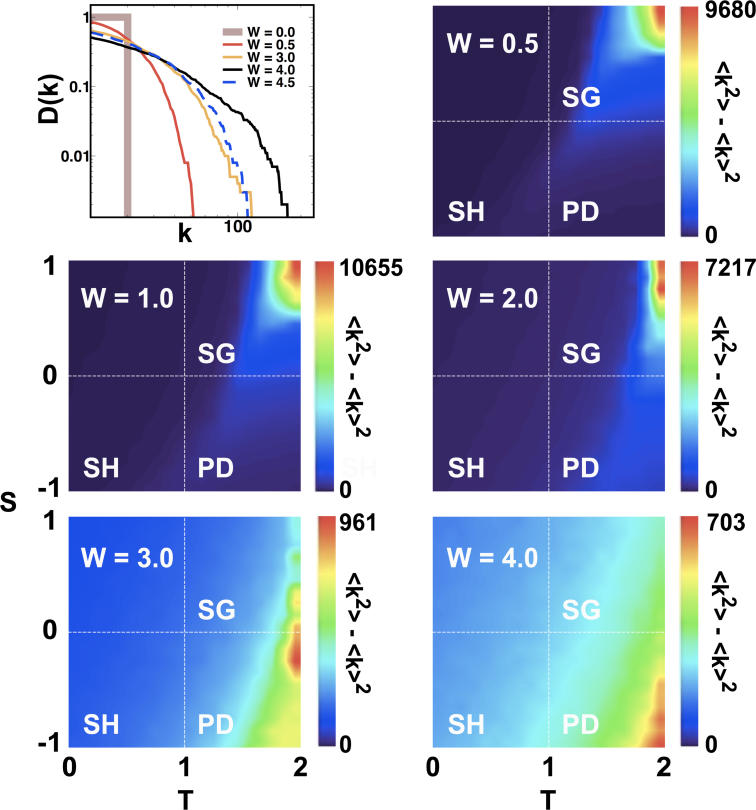
Co-Evolution and Heterogeneity We study the same social dilemmas of cooperation as in Figure 2. (Top left panel) Cumulative degree distributions (see Materials and Methods) for the most difficult challenge to cooperation in our parameter space: the PD with *T =* 2 and *S = −*1, corresponding to *b/c =* 2. Starting from a distribution exhibiting a sharp cutoff at *k_max_ = z,* as soon as *W >* 0 the distribution widens, resulting in both single-scale networks (*W* = 0.5) and broad-scale networks (*W* > 3), reflecting the increase in the overall heterogeneity of the pattern of ties in the population. (Contour plots) The amount of heterogeneity, measured in terms of the variance of the degree distribution (see Materials and Methods), depends on the underlying social dilemma and the value *W*. In other words, different challenges to cooperation lead to the evolution of different societal organization, in which simple-to-broad–scale heterogeneity develops as soon as *W ≠* 0*.* The red color corresponds to the area of the game where the conflict between strategy and topology dynamics is the strongest. For small *W,* heterogeneity is maximal for the SG and large *T*. For *W =* 4, heterogeneity is maximal for the PD with (*T =* 2, *S =* −1), in spite of the fact that cooperators wipe out defectors for all dilemmas.

For large values of the temptation *T,* the SG game leads (in well-mixed populations) to a coexistence between cooperators and defectors largely favouring the latter. Consequently, for low values of *W* and large *T,* the minority of cooperators is both unable to wipe defectors and not easily wiped out by defectors. This means that surviving cooperators will accumulate many links, leading to the most heterogeneous co-evolutionary networks encountered. Clearly, the nature of the game induces a wide diversity in the emerging topology of co-evolutionary networks. In general, one may state that the temptation to cheat (*T*) induces a more pronounced increase of the heterogeneity than the disadvantage of being cheated (*S*) [7]. Nonetheless, the overall onset of increase of heterogeneity qualitatively follows the wave of cooperation shown in Figure 2. The results shown suggest that the simple adaptive dynamics of social ties introduced here coupled with the social dilemmas accounts for the heterogeneities observed in realistic social networks [11].

### Role of Selection Pressure on Strategy and on Structure

The pairwise comparison rule [27] based on the Fermi distribution function (see Materials and Methods), has been used in updating both strategies and social ties. In general, selection may be controlled by two independent parameters: *β_e_* for strategy update and *β_a_* for adjustment of ties, both ranging from zero to infinity. Up to now we have assumed that *β_e_ = β_a_ = β*. In the following, we study the influence of changing the intensity of selection *β* on the evolution of cooperation. In a nutshell, reducing the intensity of selection will decrease the influence of the game, increasing the survival capabilities of the less fit. In Figure 5 we plot the fate of cooperators as a function of *W,* for different intensities of selection (for fixed *T =* 2 and *S =* −1, that is, a classical PD game with *b/c =* 2).

**Figure 5:**
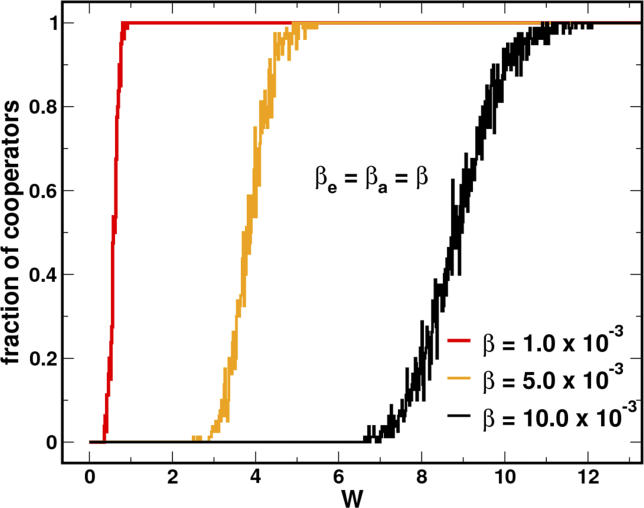
Influence of the Intensity of Selection in the PD for Different Time Scales Fraction of cooperators at the end as a function of *W* for different values of *β = β_e_ = β_a_* , *N =*10^3^ and *z =* 30. As depicted in Figure 3, the dilemma corresponds here to the PD with *b/c =* 2 (*T =* 2; *S =* −1). Decreasing values of *β* increase the viability of the less fit, which in turn makes it easier for cooperators to wipe out defectors.

Clearly, the smaller the value of *β,* the smaller *W_critical_*. Indeed, for small *W,* although cooperators are in general less fit, their survival probability increases with decreasing *β.* This increased survivability makes it more efficient for structural updating (*W >* 0) to favour assortative interactions between cooperators. As a result, the critical value of *W* at which cooperators successfully take over defectors decreases with decreasing *β*.

Let us further examine the separate role of *β_e_* and *β_a_* in the evolution of cooperation. To this end, we imposed *T =* 1 *− S* (moving along a diagonal in the PD domain, see Figure 2, from {*S =* 0;*T =* 1} to {*S =* −1;*T =* 2} corresponding to a classical PD in which *+ ∞ > b/c ≥* 2), kept a constant *W =* 2.0, and computed the survivability of cooperators and the maximum degree of connectivity *k_max_* as a function of *T*. The results are shown in Figure 6 for three different values of *β_e_,* each associated with a different panel, and, within each panel, for the three values of *β_a_* indicated. Since *W* > 0, we obtain heterogeneous populations in all cases (insets). Also, in all cases a transition from full cooperation to full defection is observed at a critical value *T_c_ ,* which depends on *β_e_* and *β_a_*. In accord with the expectations, small values of *β_e_* are associated with smoother transitions from full cooperation to full defection around *T_c_*. Furthermore, insets show that the larger the value of *β_e_ ,* the sharper is the peak of *k_max_* around *T_c_*. As *T > T_c_ ,* heterogeneity reduces to an overall baseline residual value (associated with single-scale networks), which depends on *β_e_*. Figure 6 also sheds light on the role played by *β_a_*. Overall, *β_a_* follows the effect of *β_e_ ,* shifting *T_c_* in the same direction. Indeed, the smaller the value of *β_a_ ,* i) the larger the value of *T_c_* , and ii) the smoother the transition from full cooperation to full defection. In general, with increasing *β_a_,* the fittest will be favoured, since decisions on link rewiring will become less ambiguous. As Figure 6 illustrates, however, these two parameters are not independent of each other, in spite of their similar role.

**Figure 6:**
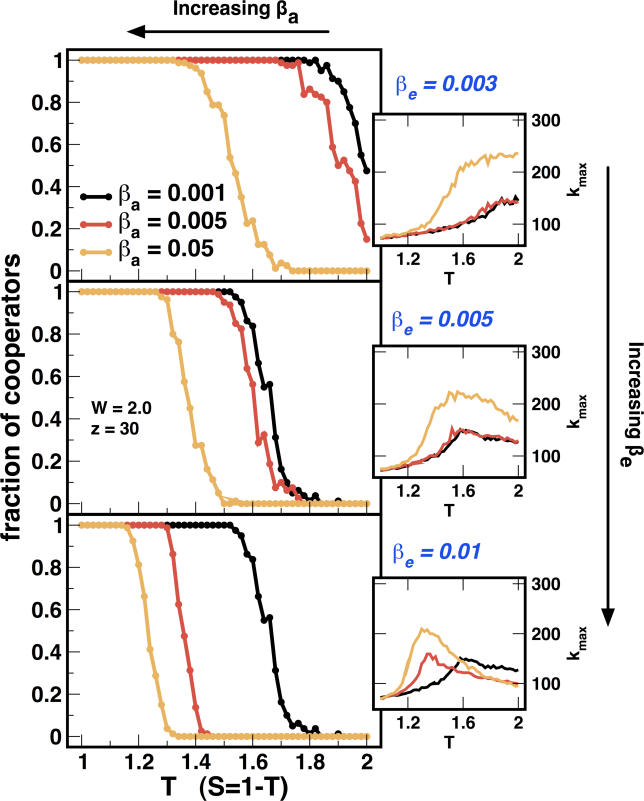
Role of Different *β* in Evolution of Cooperation Fraction of cooperators as a function of *T* for different values of *β_e_* and *β_a_*. We maintained *z =* 30 and *N =* 10^3^. The dilemma corresponds to the diagonal in the PD domain from {*S =* 0;*T =* 1} to {*S= −*1;*T =* 2} corresponding to a classical PD in which *+ ∞ > b/c ≥ 2*. The insets provide the maximum degree of the network for the different values of *T* and *S*. Both *β_e_* and *β_a_* contribute similarly (but not independently) to the evolution of cooperation. The smaller the values of *β_e_* and/or *β_a_,* the more the less fit will have a chance of survival; hence, the easier it is for cooperation to thrive.

Giving individuals control over the number and nature of their social ties, based exclusively on their immediate self-interest, leads to the emergence of long-term cooperation even in networks of high connectivity. As a consequence, “network adaptability” is required next to “social viscosity” to evolve cooperation in realistic social networks. For given values of connectivity in the population, and selection pressure, there is a critical value of *W* above which cooperators wipe out defectors. Overall, the critical value grows with increasing intensity of selection *β* and with the average connectivity *z*. The present results convey a simple yet powerful message for the evolution of cooperation: the more individuals interact, the more they must be able to promptly decide upon their partnerships for cooperation to thrive. And, of course, the less important the game is (weak selection), the less alert individuals need be.

The present work assumes that population size remains constant throughout the co-evolutionary process. This is clearly a simplification, and it remains an open problem: the role of a changing population size. Yet, the message conveyed here is powerful and shows that highly interactive, large social networks can exhibit sustained cooperation even when the benefits do not significantly exceed the costs. Finally, the process by which individuals reassess their social ties may change depending on the social context, the “game” at stake, and the species under consideration. In other words, other (perhaps more sophisticated) mechanisms may be envisaged which will certainly build upon the simple model studied here. Yet, the prospect is quite optimistic since, as shown here, simple mechanisms devoid of large information-processing requirements are capable of promoting sustained cooperation.

## Materials and Methods

### Graphs.

We place individuals on the nodes (a total of *N*) of a graph. Edges (a total of *N_E_*) represent the social ties between individuals. Graphs evolve in time as individuals change their ties. The average connectivity *z =* 2*N_E_ / N* is conserved since we do not introduce or destroy edges. At all times graphs are required to remain connected. We enforce this condition by imposing that nodes connected by a single edge cannot lose this edge. We also computed the degree of heterogeneity of the graph h = *N* ^−1^ ∑*_k_k^2^N_k_* – *z* ^2^ (where *N_k_* gives the number of vertices with *k* edges) and the cumulative degree distribution *D*(*k*) = *N* ^−1^ 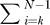 *N_i_*. Since *D*(*k*) = 0 for *k* > *k_max_ ,* with *k_max_* the maximum value of the connectivity of a graph, both *h* and *k_max_* provide simple measures of the heterogeneity of a graph.

### Evolution of strategies.

Whenever *W >* 0, evolution of strategy and structure proceed together under asynchronous updating. Choice of type of update event depends on *W;* assuming (without loss of generality) *τ_e_* = 1, a strategy update event is chosen with probability (1 + *W*)^−1^, a structural update event being selected otherwise. A strategy update event is defined in the following way, corresponding to the so-called pairwise comparison rule [27]: one node *A* is chosen at random and another node *B* is chosen randomly among *A*'s first neighbours. The individuals *A* and *B* interact with all their neighbours (those directly connected to them by edges) according to the social dilemma specified. As a result, they accumulate total payoffs Π(*A*) and Π(*B*)*,* respectively. The strategy of *B* replaces that of *A* with a  probability given by the (Fermi distribution) function *p* = [1 + *e* ^−*β*[Π(*B*) − Π(*A*)]^]^ −1^. The value of *β* (≥0), which plays the role of an inverse temperature in statistical physics, controls here the intensity of selection (*β* → 0 leads to neutral drift whereas *β* → ∞ leads to the so-called imitation dynamics, often used to model cultural evolution).

### Computer Simulations.

We start from a homogeneous random graph [28], in which all nodes have the same number of edges (*z*), randomly linked to arbitrary nodes. Population size is *N =* 10^3^ and average connectivities *z =* 10, 20, 30, and 40 (the value *z =* 30 used in Figure 2 reflects the mean value of the average connectivities reported in [13] for socials networks). We start with 50% of cooperators randomly distributed in the population. We run 100 independent simulations for each set of parameters (*T, S, W*) and compute the fraction of times that evolution stopped at 100% cooperation. If, after 10^8^ generations, the population has not converged to an absorbing state, we take as the final result the average fraction of cooperators in the population over the last 1,000 generations. Indeed, especially for the SG, the time for reaching an absorbing state may be prohibitively large [27]. At the end of each evolution we also computed the heterogeneity *h* and maximal connectivity *k_max_* associated with the final graph and the cumulative degree distribution, which are on the basis of the results plotted in Figure 3. We have confirmed that our results are valid for *N >* 500.

## Abbreviations

PD: prisoner's dilemma
SG: snowdrift game
SH: stag-hunt game
